# Impact of Pulmonary Ligament Resection in Upper Lobectomies: A Multicenter Matched Cohort Study

**DOI:** 10.3390/jcm13226950

**Published:** 2024-11-18

**Authors:** Alessio Campisi, Andrea Dell’Amore, Wentao Fang, Gabriella Roca, Stefano Silvestrin, Samuele Nicotra, Yang Chen, Piotr Gabryel, Magdalena Sielewicz, Cezary Piwkowski, Eleonora La Rocca, Alexandro Patirelis, Vincenzo Ambrogi, Riccardo Giovannetti, Federico Rea, Maurizio Infante

**Affiliations:** 1 Department of Thoracic Surgery, Shanghai Chest Hospital, Shanghai Jiao Tong University, Shanghai 200030, China; vwtfang@hotmail.com (W.F.); cyang0952@163.com (Y.C.); 2 Thoracic Surgery Unit, Cardiovascular and Thoracic Department, University and Hospital Trust-Ospedale Borgo Trento, 37126 Verona, Italy; giovannetti.riccardo@gmail.com (R.G.); maurizio.infante@aovr.veneto.it (M.I.); 3 Department of Cardiothoracic Surgery and Vascular Sciences, Padua University Hospital, University of Padua, 35128 Padua, Italy; andrea.dellamore@unipd.it (A.D.); gabriella.roca@studenti.unipd.it (G.R.); stefano.silvestrin@studenti.unipd.it (S.S.); samuele.nicotra@aopd.veneto.it (S.N.); federico.rea@unipd.it (F.R.); 4 Department of Thoracic Surgery, Poznan University of Medical Sciences, 60-569 Poznan, Poland; piotrgabryel@gmail.com (P.G.); magda.sielewicz@gmail.com (M.S.); cezary_p@hotmail.com (C.P.); 5 Thoracic Surgery Unit, Tor Vergata University Polyclinic, 00133 Rome, Italy; eleonora.larocca@virgilio.it (E.L.R.); alexandro.patirelis@hotmail.it (A.P.); vincenzo.ambrogi@uniroma2.it (V.A.)

**Keywords:** lung cancer, minimally invasive, lobectomy, pulmonary ligament

## Abstract

**Background**: Division of the pulmonary ligament is standard in lower lobectomies, but its application in upper lobectomies remains controversial due to potential complications like atelectasis and bronchial kinking. This retrospective matched cohort study aimed to evaluate the efficacy and safety of ligament resection in upper lobectomies for oncological purposes. **Methods**: From January 2015 to December 2020, 988 patients who underwent minimally invasive upper lobectomies across multiple centers were identified. They were categorized into ligament resection and no ligament resection groups, with propensity score matching (PSM) to minimize confounding factors. Endpoints included operative time, pleural effusion, complications (frequency and Clavien–Dindo scores), chest drainage removal, length of stay, pleural space, collapse rate, and bronchial kinking. **Results**: Following PSM, 276 patients were included in each group, with no significant differences in baseline characteristics. Ligament resection correlated with longer operative times, increased lymphadenectomy sampling at station #9 (*p* < 0.001), and a bigger change in the bronchial angle (*p* < 0.001). No statistically significant differences were observed for the other endpoints. **Conclusions**: Ligament resection during upper lobectomy may impact the bronchial angle without immediate postoperative outcome changes. Further research is necessary to comprehensively assess the risks and benefits of ligament resection in upper lobectomies for neoplastic disease.

## 1. Introduction

Lobectomy stands as the cornerstone treatment for early-stage non-small-cell lung cancer (NSCLC) [1]. The division of the pulmonary ligament is a routine procedure in lower lobectomies to isolate and section the inferior pulmonary vein. Conversely, it is not technically essential for upper lobectomies. Indeed, it does not facilitate the identification and isolation of the involved hilar structures. Furthermore, concerning lymphadenectomy, its necessity is questionable, as the specific lobar lymphatic drainage is delineated by stations 2R and 4R for the right upper lobe and 4L, 5, and 6 for the left upper lobe [2].

Traditionally, many thoracic surgeons have performed the division of the inferior pulmonary ligament for upper lobectomies under the assumption that it enhances mobility, thus aiding in the re-expansion of the residual parenchyma to fill the entire pleural cavity and mitigate the development of pleural effusion, thus reducing the duration of pleural drainage, shortening hospitalization, and lowering the risk of postoperative infections. Nevertheless, subsequent studies have cast doubt on these assumptions [3,4,5,6]. Instead, this procedure may result in postoperative complications such as bronchial kinking, leading to residual parenchymal atelectasis and respiratory dysfunction [3,4,5,6]. Presently, there exists no consensus on the utility and associated benefits of dissecting the pulmonary ligament during upper lobectomies for neoplasms.

Our study aimed to evaluate whether there is an increased occurrence of intraoperative, perioperative, and postoperative complications linked to the division of the ligament during upper lobectomies and whether this practice confers advantages.

## 2. Materials and Methods

This is a retrospective analysis of consecutive patients who underwent upper lobectomies (right and left) for neoplastic disease in our centers from January 2015 to December 2020 (Supplementary File S1 shows the number of patients collected from each center before and after PSM). The institutional review board of the coordinating hospital approved the study (N° 2528-CESC). The study was performed in line with the principles of the Declaration of Helsinki. The paper was written according to the STROCSS criteria (strengthening the reporting of cohort studies in surgery). The checklist is provided as Supplementary File S2 [7].

Preoperative radiological and invasive staging procedures were conducted in accordance with prevailing protocols.

All patients who underwent minimally invasive video-assisted upper lobectomies were included in the study. Patients were divided into two groups: the ligament resection group and the no ligament resection group.

In both groups, surgeries were conducted identically except for the ligament resection procedure. Typically, ligament resection is carried out using electrocautery or energy devices, with concurrent lymphadenectomy of station #9. In the group where ligament resection was not performed, lymphadenectomy of station #9 is typically omitted unless clinically indicated. Lobectomy, as well as ligament division, is typically performed according to the surgeon’s preference, utilizing either a one-, two-, or three-port approach. Vessel and bronchial dissection are carried out based on individual surgical judgment and technique suitability. Lymphadenectomy follows the guidelines outlined by the European Society of Thoracic Surgeons (ESTS) in cases of ligament resection [2]. In the non-ligament resection group, lobe-specific systematic nodal dissection was performed [8]; however, in cases of clear or highly suspicious involvement of station #9 lymph nodes, these were removed without transecting the ligament.

Exclusion criteria were as follows: Video-Assisted Thoracoscopic Surgery (VATS) non-anatomical lung resections; VATS segmentectomies; VATS lower, middle, and bi-lobectomies; VATS lobectomies associated with chest wall or diaphragm resections; sleeve resections (bronchial and vascular); previous ipsilateral surgery; conversion to open surgery; neoadjuvant chemotherapy (ChT); neoadjuvant thoracic radiotherapy (RT); previous breast cancer treatment; surgery performed for tumors other than NSCLC; and incomplete patient data.

The aim of this retrospective case–control study was to explore the utility and safety of ligament resection in upper lobectomies for oncological purposes. The endpoints were

- perioperative results: operative time (min), pleural effusion (mL), complications (frequency and scores [9]), chest drainage removal (days) and length of stay, pleural space (defined as the presence of >20% pneumothorax or a 3 cm gap between visceral pleura and chest wall on a chest radiograph at the I postoperative day—POD [10]) and collapse rate (calculated on a chest X-ray by taking the difference between the preoperative baseline area and the actual postoperative area of the remaining lungs, dividing that difference by the preoperative area, and then multiplying the result by 100 to express it as a percentage).
- long-term results (after at least 3 months from the surgery): changes in the bronchial angle (defined as the convex angle formed between the axis of the trachea and the angles of the intermedius bronchus on the right side and the inferior bronchus on the left side), long-term complications, diaphragmatic paralysis (calculated quantitatively using the distance between the highest point of the diaphragm and the apex of the chest before and after surgery and defined as more than 30% [11] and qualitatively using ultrasound, fluoroscopy, or electrodiagnostic studies).

For the purpose of the study, patient records were evaluated solely for endpoints, and oncological long-term results were not analyzed and thus omitted.

We examined our database for the general, perioperative, and oncological characteristics of the patients. TNM staging was determined according to the 8th edition of the *AJCC Cancer Staging Manual* [12].

### Statistical Analysis

Analyses were conducted with IBM SPSS Statistics (IBM Corp. Released 2017. IBM SPSS Statistics for Windows, Version 25.0. Armonk, NY: IBM Corp). Continuous variables are expressed as a mean ± standard deviation (SD) or median and range when appropriate, categorical variables are expressed as numbers and percentages.

In order to minimize the lack of randomization, a propensity score-matched (PSM) analysis was used to mitigate the confounding factors. We employed a nearest neighbor matching algorithm without replacement, using a caliper of 0.02 to select the most appropriately matched pairs. Matching variables included sex, age, body mass index (BMI), smoking habits, lung function (Forced Expiratory Volume after 1 s—FEV1; forced vital capacity—FVC), and Charlson Comorbidity Index scores [13]. Standardized mean difference (SMD) was defined as the difference in the means of the 2 groups divided by the standard deviation. An SMD  <  0.2 was considered to demonstrate an acceptable balance.

The significance level was set at 5% (*p* = 0.05). Continuous variables were first assessed for normality using the Shapiro–Wilk test. Based on this assessment, the two groups were compared using the unpaired t-test for normally distributed data or the Mann–Whitney U test for non-normally distributed data. Discrete or categorical data were compared using the chi-square test or Fisher’s exact test, as appropriate. A multivariable Cox proportional hazards model was constructed based on hypothesized clinical relevance and the results of univariable analysis (*p* < 0.2).

## 3. Results

In total, 988 patients, including 490 patients in the no ligament resection group and 498 in the ligament resection group, met the study criteria. General characteristics of the patients before and after PSM are shown in Table 1. Patients differed for all the characteristics except for FVC. After PSM, a total of 276 patients were included in each group.

Table 2 summarizes perioperative characteristics of the matched cohorts, and there were no significant differences observed between the two groups except for surgery time which was longer in the ligament resection group (*p* < 0.001), and the lymph node station #9, which was harvested only in the ligament resection group.

Table 3 shows the early and long-term complications and histological and short-term oncological results. The only difference was reported in bronchial angle between the two groups (*p* < 0.001), with the ligament resection group showing a bigger change in the angle compared to the no ligament resection group. There was no significant difference in 1-year survival rates between the two groups (*p* = 0.154).

As a collateral analysis, we examined the different outcomes between right and left upper lobectomies and they are shown in Table 4. Ligament resection was associated with significantly longer surgery times compared to cases without ligament resection on the right side (*p* < 0.001), but not on the left side (*p* = 0.411). Additionally, there was a higher incidence of pleural space presence in the non-resection group, which was particularly evident in right-sided lobectomies (13.3% vs. 6.4%, *p* = 0.033). Bronchial angle changes were significant in both sides (*p* = 0.001 and 0.016, respectively).

Table 5 presents the results of linear regression analyses for complications. In multivariable analysis, surgery time emerged as the sole independent risk factor (*p* = 0.005, HR = 1.007, 95% CI = 1.002–1.011), indicating that for each additional minute of surgery time, the risk of complications increased by approximately 0.7%.

None of the factors examined were found to be independently predictive of pleural space occurrence (Table 6).

## 4. Discussion

Our multicenter retrospective cohort study aimed to evaluate the efficacy and safety of pulmonary ligament resection in upper lobectomies for neoplastic disease. The debate over whether to perform ligament resection in upper lobectomies has persisted due to conflicting evidence regarding its benefits and potential complications [3,4,5,6,14].

Our results showed that ligament resection correlated with a longer median operative time and a higher frequency of lymphadenectomy of station #9. Clearly, the mean operative time significantly increases in the group with pulmonary ligament sectioning. Pulmonary ligament sectioning is not particularly time-consuming but may require instruments to be repositioned to correctly visualize anatomical structures, mobilize the lower lobe without injuring it, and avoid injury to nearby structures (esophagus, aorta, lower pulmonary vein). We did not expect such a significant difference (20 min median) in operative times, and this discrepancy may be caused by multiple factors, such as tumor stage and the extent of lymphadenectomy performed. Regarding station #9 lymphadenectomy, it was predictable that it would be significant in favor of the ligament sectioning group since the two events are practically sequential. However, it is noteworthy that two patients with positivity in the ligament resection group already had multistation mediastinal positive lymph nodes and would have received adjuvant treatment regardless, so the station 9 positivity likely did not affect their prognosis. In fact, as recently proposed by Yazgan et al. [15], exploring station 9 in upper lobectomies does not significantly impact disease staging and survival.

We observed no significant differences in complication incidence or severity between the two groups. However, it is noteworthy that the ligament resection group encountered two severe complications, including one instance of esophageal perforation resulting in in-hospital mortality and another case of lobar torsion necessitating pneumonectomy. Despite the infrequency of such severe complications within the ligament resection group, their occurrence prompts careful consideration. The case of esophageal perforation leading to in-hospital death underscores the potential risks inherent in this surgical approach. Although esophageal injury during lung lobectomy is uncommon, its consequences can be grave, encompassing infection, sepsis, and fatality. Similarly, the instance of lobar torsion requiring pneumonectomy raises concerns regarding the possibility of anatomical distortion or disruption following ligament resection.

On the other hand, there were no significant differences in residual pleural cavity, collapse rate, drained fluid volume, or PAL. These results are in line with our idea that the assumption that ligament resection may improve lung re-expansion with better pleural cavity re-occupation with lower pleural effusion is just historical and confirms previous literature [3,4,5,6].

The other main issue of ligament resection is bronchial angle change. Several studies have demonstrated how ligament sectioning may increase the risk of bronchial angle modifications concerning the tracheal axis, bronchial torsion, consequent airflow alteration, and residual parenchymal atelectasis leading to respiratory dysfunction [3]. Conversely, many studies do not highlight significant bronchial anatomy changes [7]. In our study, bronchial angle modification after ligament sectioning was significantly different; in fact, in patients after resection, angle modification was bigger (135.50 vs. 124.0, *p* < 0.001), despite having no clinical relevance. Regardless, the significant difference in bronchial angle between the two groups warrants attention. In fact, as previously reported by Bu et al. [3], preservation of the pulmonary ligament during VATS lobectomy might have an impact on lung function and lung volume. Unfortunately, we lacked data on postoperative lung function for all patients, primarily due to the nature of the hospital being a referral center and patients returning to their hometowns post-surgery.

The comparison of outcomes between right and left upper lobectomies, with and without ligament resection, sheds light on several important factors influencing postoperative recovery and complications. Notably, there was a statistically significant difference in surgery time, with right-sided procedures taking longer when the ligament was transected. While the exact explanation is challenging, it may be attributed to factors such as the size of the lung and the presence of three lobes, necessitating additional maneuvers. Additionally, the need to consistently verify the presence of the lower vein on the left side, which may occasionally be omitted on the right side if the middle lobe vein is clearly visualized, could contribute to the observed difference. Pleural space-related parameters also revealed intriguing trends; the incidence of pleural space was significantly higher in right upper lobectomies without ligament resection, despite the idea that the left upper lobe may leave a bigger space behind. Our result could be explained by two factors: firstly, on the left side, cardiac obstruction might favor the ascent of the residual lobe and space occupation; secondly, anesthetic maneuvers and postoperative analgesia usage (resulting in persistent peristaltic slowing) frequently cause gastric bubble swelling, leading to elevated left diaphragm and consequently quicker re-occupation of pleural space. The observed differences in bronchial angles within the entire cohort remained consistent when considering each side individually. This suggests that the impact of ligament resection on bronchial angle alteration is uniform across both the right and left sides.

Despite its strengths, this study has limitations to consider. The sample size, while substantial, may limit generalizability, as the study was conducted at specific centers that may not fully represent the broader patient population. Additionally, a few centers contributed patients to only one of the groups, which may affect the generalizability of the results (a sub-analysis of individual center outcomes is reported in Supplementary File S3). Its retrospective nature introduces inherent biases, such as selection bias and incomplete data capture, despite efforts to minimize bias through propensity score matching. Procedures were performed by multiple surgeons with individual techniques and preferences, potentially confounding the results. No standard protocols were used for postoperative chest drainage management and patient discharge, which may influence our results. Moreover, we do not have the number of patients who were excluded because they did not fit the inclusion criteria. TNM staging was not used in the PSM analysis, which may influence the surgical time results, as more advanced cases may have prolonged the surgery (a sub-analysis of early versus advanced tumor stage outcomes is reported in Supplementary File S4). The study’s follow-up duration may not fully capture long-term oncological outcomes. Finally, all patients in our study underwent VATS lobectomies, which inherently differ from open surgical approaches. VATS involves less extensive manipulation of the thoracic cavity, potentially influencing outcomes differently compared to open surgery [16].

Overall, while ligament resection in upper lobectomy remains a subject of debate, our study found no significant differences in the analyzed outcomes except for the bronchial angle. However, this difference in the bronchial angle does not represent a higher risk of postoperative atelectasis. Therefore, individualized decision-making based on patient characteristics and surgeon expertise remains important in determining the appropriateness of ligament resection in upper lobectomy.

## Figures and Tables

**Table 1:** General characteristics of pre-matched patients.

	Before PSM			After PSM		
	No Ligament Resection (n = 490)	Ligament Resection (n = 498)	*p*-Value	No Ligament Resection (n = 276)	Ligament Resection (n = 276)	*p*-Value
Age	63.0 (56.0–71.0)	69.0 (63.0–74.0)	<0.001 *a	68.0 (61.0–72.75)	68.0 (60.0–73.0)	0.889 a
Sex			0.005 *b			0.087 b
Male	232 (47.3%)	280 (56.2%)		161 (58.3%)	141 (51.1%)	
Female	258 (52.7%)	218 (43.8%)		115 (41.7%)	135 (48.9%)	
BMI	23.96 (21.71–26.35)	25.91 (23.12–28.41)	<0.001 *a	25.125 (22.31–27.73)	25.41 (22.95–28.38)	0.214 a
Smoking			<0.001 *b			0.280 b
Current	107 (21.8%)	128 (25.7%)		91 (33.0%)	74 (26.8%)	
Never	283 (57.8%)	104 (20.9%)		89 (32.2%)	95 (34.4%)	
Previous	100 (20.4%)	266 (53.4%)		96 (33.8%)	107 (38.8%)	
FEV1 (%)	95.76 (84.0–107.0)	92.0 (77.0–106.25)	0.001 *a	98.21 ± 19.72	95.10 ± 21.25	0.313 c
FVC (%)	96.10 (86.2–108.0)	98.0 (87.0–111.0)	0.204 a	98.10 (88.0–110.0)	99.0 (88.0–112.0)	0.622 a
Comorbidities (pts)	265 (54.1%)	403 (80.9%)	<0.001 *b	194 (70.3%)	199 (72.1%)	0.638 b
Charlson Comorbidity Index	3.0 (2.0–4.0)	4.0 (3.0–5.0)	<0.001 *a	4.0 (3.0–5.0)	4.0 (3.0–5.0)	0.564 a

Notes: Data are presented as median (P25–P75) or n (%). * *p* < 0.05. a: Mann–Whitney U test; b: chi-square test; c: *t*-test. Abbreviations: PSM: propensity score matching; BMI, body mass index; FEV1, Forced Expiratory Volume after 1 s; FVC, forced vital capacity; pts, patients.

**Table 2:** Perioperative characteristics of patients.

	No Ligament Resection (n = 276)	Ligament Resection (n = 276)	*p*-Value
Side			0.861 b
Right	173 (62.7%)	171 (62.0%)	
Left	103 (37.3%)	105 (38.0%)	
Surgery time (minutes)	120.0 (100.0–151.50)	140.0 (110.0–180.0)	<0.001 *a
Estimated blood loss (mL)	100.0 (50.0–100.0)	100.0 (50.0–100.0)	0.068 a
Lymph node (number)	10.0 (7.0–12.0)	9.0 (7.0–12.0)	0.657 a
Lymph node station (number)	5.0 (5.0–6.0)	5.0 (4.0–6.0)	0.945 a
Lymph node station#9 harvested (yes)	0 (0.0%)	127 (46.0%)	<0.001 *
Pleural space (yes)	30 (10.9%)	19 (6.9%)	0.100 b
Pleural space (mm)	40.0 (34.75–55.5)	35.0 (30.0–44.0)	0.096 a
Collapse rate (%)	7.0 (4.0-10.0)	7.0 (5.0–10.0)	0.361 a
POD1 effusion (mL)	250.0 (250.0–257.50)	250.0 (200.0–357.50)	0.155 a
POD2 effusion (mL)	150.0 (150.0–250.0)	150.0 (100.0–300.0)	0.620 a
POD3 effusion (mL)	150.0 (150.0–168.75)	150.0 (100.0–200.0)	0.520 a
Chest drainage duration (days)	3.0 (2.0–4.0)	3.0 (2.0–5.0)	0.133 a
Discharge with drainage	0 (0.0%)	0 (0.0%)	-
Postoperative bronchoscopy abnormalities (yes)	1 (0.4%)	0 (0.0%)	0.317 b
Bronchial kinking	0 (0.0%)	1 (0.4%)	0.317 b
LOH (days)	5.0 (4.0–7.0)	5.0 (3.0–7.0)	0.203 a
In-hospital mortality	0 (0.0%)	1 (0.4%)	0.317 b
30-day mortality	0 (0.0%)	2 (0.7%)	0.157 b
90-day mortality	0 (0.0%)	3 (1.1%)	0.082 b

Notes: Data are presented as mean (±SD) median (P25–P75) or n (%). * *p* < 0.05. a: Mann–Whitney U test; b: chi-square test. Abbreviations: pts, patients; POD, postoperative day; LOH, length of hospital stay.

**Table 3:** Early and long-term complications and histological and short-term oncological results.

	No Ligament Resection (n = 276)	Ligament Resection (n = 276)	*p*-Value
Early complications (pts)	38 (13.8%)	52 (18.8%)	0.107 a
Number of complications			0.272 a
one	33 (12.0%)	45 (16.3%)	
two	5 (1.8%)	7 (2.5%)	
Early complications (type)			0.379 a
Pneumonia	8 (2.9%)	3 (1.1%)	
ARDS	0 (0.0%)	2 (0.7%)	
Atrial fibrillation	14 (5.1%)	24 (8.4%)	
PAL	13 (4.7%)	12 (4.3%)	
Pneumothorax	0 (0.0%)	1 (0.4%)	
Atelectasis	1 (0.4%)	2 (0.7%)	
Anemia	0 (0.0%)	2 (0.7%)	
Lung hemorrhage	0 (0.0%)	1 (0.4%)	
Delirium	0 (0.0%)	1 (0.4%)	
Kidney failure	0 (0.0%)	1 (0.4%)	
Bleeding	1 (0.4%)	1 (0.4%)	
RLN paralysis	2 (0.7%)	3 (1.1%)	
Pulmonary infarction	0 (0.0%)	1 (0.4%)	
Diaphragmatic elevation	4 (1.4%)	4 (1.4%)	
Esophageal injury	0 (0.0%)	1 (0.4%)	
Treatment of early complication			0.556 a
Observation	4 (1.4%)	3 (1.1%)	
Medical therapy	18 (6.5%)	20 (7.2%)	
Chest drainage insertion	0 (0.0%)	1 (0.4%)	
Completion pneumonectomy	0 (0.0%)	1 (0.4%)	
Endoscopic approach	1 (0.4%)	3 (1.1%)	
Reoperation	1 (0.4%)	1 (0.4%)	
ABPP for PAL	14 (5.1%)	23 (8.3%)	
PAL (pts)	14 (5.1%)	24 (8.7%)	0.093 a
Diaphragmatic elevation (yes)	4 (1.4%)	4 (1.4%)	1.000 b
Chest drainage reinsertion (yes)	0 (0.0%)	1 (0.4%)	1.000 b
Clavien–Dindo Classification			0.311 a
Grade 1	6 (2.2%)	6 (2.2%)	
Grade 2	30 (10.9%)	36 (13.0%)	
Grade 3A	1 (0.4%)	4 (1.4%)	
Grade 3B	1 (0.4%)	2 (0.7%)	
Grade IVA	0 (0.0%)	3 (0.7%)	
Grade IVB	0 (0.0%)	0 (0.0%)	
Grade V	0 (0.0%)	1 (0.4%)	
Late complications (pts)	26 (9.4%)	27 (9.8%)	0.885 a
Late bronchial kinking	0 (0.0%)	0 (0.0%)	-
Late complications (type)			0.958 a
Arrhythmia	18 (6.5%)	18 (6.5%)	
RLN palsy	2 (0.7%)	3 (1.1%)	
Diaphragmatic paralysis	4 (1.4%)	5 (1.8%)	
Chronic cough	2 (0.7%)	1 (0.4%)	
Treatment of late complications			0.710 a
Observation	4 (1.4%)	5 (1.8%)	
Medical therapy	20 (7.2%)	19 (6.9%)	
Logopedic therapy	1 (0.4%)	3 (1.1%)	
Thyroplasty	1 (0.4%)	0 (0.0%)	
Bronchial angle (°)	135.5 (122.25–148.0)	124.0 (107.0–145.0)	<0.001 *c
Diaphragmatic paralysis (yes)	4 (1.4%)	5 (1.8%)	1.000 b
Histology			0.740 a
adenocarcinoma	220 (79.7%)	222 (80.4%)	
SSC	38 (13.8%)	34 (12.3%)	
Large cell carcinoma	8 (2.9%)	12 (4.3%)	
Adenosquamous	10 (3.6%)	8 (2.9%)	
Tumor size (cm)	2.58 ± 1.23	2.77 ± 1.35	0.074 c
Tumor status			0.254 a
pT1a	13 (4.7%)	16 (5.8%)	
pT1b	88 (31.9%)	90 (32.6%)	
pT1c	78 (28.3%)	55 (19.9%)	
pT2a	67 (24.3%)	69 (25.0%)	
pT2b	14 (5.1%)	23 (8.3%)	
pT3	14 (5.1%)	20 (7.2%)	
pT4	2 (0.7%)	3 (1.1%)	
Lymph node status			0.311 a
N0	250 (90.6%)	240 (87.0%)	
N1	14 (5.1%)	16 (5.8%)	
N2	12 (4.3%)	20 (7.2%)	
Lymph node station #9 positive	0 (0.0%)	2 (0.7%)	0.499 b
TNM staging (8th edition)			0.116 a
IA	176 (63.8%)	149 (54.0%)	
IB	52 (18.8%)	56 (20.3%)	
IIA	11 (4.0%)	19 (6.9%)	
IIB	19 (6.9%)	27 (9.8%)	
IIIA	17 (6.2%)	20 (7.2%)	
IIIB	1 (0.4%)	5 (1.8%)	
1-year survival	274 (99.3%)	270 (97.8%)	0.154 a

Notes: Data are presented as median (P25–P75) or n (%). * *p* < 0.05. a: chi-square test; b: Fisher’s exact test; c: Mann–Whitney U test. Abbreviations: pts, patients; ARDS, acute respiratory distress syndrome; PAL, prolonged air leak; RLN, recurrent laryngeal nerve; ABPP, autologous blood patch pleurodesis; SSC, squamous cell carcinoma.

**Table 4:** Different outcomes between right and left upper lobectomies.

	No Ligament Resection Right (n = 169) Left (n = 103)	Ligament Resection Right (n = 167) Left (n = 105)	*p*-Value
Surgery time (minutes)			
Right	120.0 (99.50–145.0)	140.0 (110.0–180.0)	<0.001 *a
Left	136.94 ± 44.68	142.28 ± 48.64	0.411 b
Pleural space (yes)			
Right	23 (13.3%)	11 (6.4%)	0.033 *c
Left	7 (6.8%)	8 (7.6%)	0.819 c
Pleural space (mm)			
Right	43.0 (38.0–61.0)	35.0 (30.0–45.0)	0.077 a
Left	35.71 ± 4.23	37.62 ± 5.78	0.484 b
Collapse rate (%)			
Right	7.0 (4.0–10.0)	7.0 (5.0–11.0)	0.437 a
Left	7.0 (4.0–10.0)	7.0 (5.0–10.0)	0.639 a
POD1 effusion (mL)			
Right	250.0 (250.0–250.0)	250.0 (200.0–400.0)	0.072 a
Left	250.0 (160.0–300.0)	250.0 (150.0–350.0)	0.915 a
POD2 effusion (mL)			
Right	150.0 (150.0–240.0)	150.0 (100.0–300.0)	0.598 a
Left	150.0 (100.0–275.0)	170.0 (100.0–300.0)	0.177 a
POD3 effusion (mL)			
Right	150.0 (150.0–162.50)	150.0 (100.0–200.0)	0.241 a
Left	150.0 (50.0–200.0)	150.0 (100.0–200.0)	0.700 a
Chest drainage duration (days)			
Right	3.0 (2.0–4.5)	3.0 (2.0–5.0)	0.416 a
Left	3.0 (2.0–4.0)	3.0 (2.0–5.0)	0.154 a
Early complications (pts)			
Right	25 (14.5%)	31 (18.1%)	0.356 c
Left	13 (12.6%)	21 (20.0%)	0.150 c
PAL (pts)			
Right	12 (6.9%)	16 (9.4%)	0.412 c
Left	2 (1.9%)	8 (7.6%)	0.056 c
Diaphragmatic elevation (yes)			
Right	4 (2.3%)	3 (1.8%)	1.000 d
Left	0 (0.0%)	1 (1.0%)	1.000 d
Bronchial angle (°)			
Right	135.37 ± 24.67	126.84 ± 23.67	0.001 *b
Left	133.11 ± 16.08	126.49 ± 22.64	0.016 *b
Diaphragmatic paralysis (yes)			
Right	4 (2.3%)	4 (2.3%)	1.000 d
Left	0 (0.0%)	1 (1.0%)	1.000 d

Notes: Data are presented as mean (±SD) median (P25–P75) or n (%). * *p* < 0.05. a: Mann–Whitney U test; b: *t*-test; c: chi-square test; d: Fisher’s exact test. Abbreviations: POD, postoperative day; pts, patients; PAL, prolonged air leak.

**Table 5:** Linear regression analyses for complications.

	Univariable Analysis	Multivariable Analysis		
Variable	*p*-Value	HR	95% CI	*p*-Value
Gender	0.684	-	-	-
Age	0.010	1.020	0.990–1.052	0.191
BMI	0.789	-	-	-
Smoking habits	0.978	-	-	-
CCI	0.003	1.140	0.952–1.365	0.154
Side upper lobectomy	0.984	-	-	-
Surgery time (min)	0.001	1.007	1.002–1.011	0.005 *

Notes: * *p* < 0.05. Abbreviations: BMI, body mass index; CCI, Charlson Comorbidity Index.

**Table 6:** Linear regression analyses for pleural space.

	Univariable Analysis	Multivariable Analysis		
Variable	*p*-Value	HR	95% CI	*p*-Value
Gender	0.338	-	-	-
Age	0.290	-	-	-
BMI	0.044	1.049	0.986–1.115	0.128
Smoking habits	0.242	-	-	-
CCI	0.232	-	-	-
Side upper lobectomy	0.286	-	-	-
Surgery time (min)	0.188	1.003	0.997–1.008	0.356

Notes: *p* < 0.05. Abbreviations: BMI, body mass index; CCI, Charlson Comorbidity Index.

## Data Availability

The data underlying this article will be shared upon reasonable request to the corresponding author.

## Supplementary Materials

The following supporting information can be downloaded at https://www.mdpi.com/article/10.3390/jcm13226950/s1: Supplementary File S1: The number of patients included from each center; Supplementary File S2: The STROCCS checklist; Supplementary File S3: A sub-analysis of individual center outcomes; Supplementary File S4: A sub-analysis of early versus advanced tumor stage outcomes.

## Abbreviations and Acronyms

ABPP	autologous blood patch pleurodesis
AJCC	American Joint Committee on Cancer
ARDS	acute respiratory distress syndrome
BMI	body mass index
CCI	Charlson Comorbidity Index
ChT	chemotherapy
CT	computed tomography
ESTS	European Society of Thoracic Surgery
FEV1	Forced Expiratory Volume after 1 s
FVC	forced vital capacity
LOH	length of hospital stay
ml	milliliters
min	minutes
MRI	magnetic resonance imaging
NSCLC	non-small-cell lung cancer
PAL	prolonged air leak
PET	positron emission tomography
POD	postoperative day
PSM	propensity score matching
pts	patients
RLN	recurrent laryngeal nerve
RT	radiotherapy
SMD	standardized mean difference
SSC	squamous cell carcinoma
STROCSS	strengthening the reporting of cohort studies in surgery
TNM	tumor, lymph node, metastasis
VATS	Video-Assisted Thoracoscopic Surgery
