# Supplementary material for: Impact of Pulmonary Ligament Resection in Upper Lobectomies: A Multicenter Matched Cohort Study
Source: J Clin Med. 2024 Nov 18;13(22):6950. doi: 10.3390/jcm13226950 (PMC11594900; doi:10.3390/jcm13226950)
Supplement: Supplementary file 1 [file jcm-13-06950-s001.zip › ligament jcm/Supplementary File.1.docx]

| **Supplementary File 1. The number of patients included from each center.** | |
| --- | --- |
| **Center** | **Patients** |
| Shanghai Chest Hospital | 306 |
| Padua University Hospital | 326 |
| Poznan University of Medical Sciences | 138 |
| Ospedale Borgo Trento - Verona | 153 |
| Tor Vergata University Polyclinic | 65 |
