# Supplementary material for: Impact of Pulmonary Ligament Resection in Upper Lobectomies: A Multicenter Matched Cohort Study
Source: J Clin Med. 2024 Nov 18;13(22):6950. doi: 10.3390/jcm13226950 (PMC11594900; doi:10.3390/jcm13226950)
Supplement: Supplementary file 1 [file jcm-13-06950-s001.zip › Supplementary file 4.docx]

| Supplementary Table S7. Different outcomes between early (stage I and II) and advanced stages (stage III). | | | |
| --- | --- | --- | --- |
|  | NO LIGAMENT RESECTION  early (n=258)  advanced (n=18) | LIGAMENT RESECTION  early (n=251)  advanced (n=25) | p Value |
| Surgery Time (minutes)  early  advanced | 120.0 (100.0-150.0)  140.0 (113.75-180.0) | 140.0 (110.0-180.0)  130.0 (112.50-167.50) | <0.001*a  0.666a |
| Pleural space (yes)  early  advanced | 28 (10.9%)  2 (11.1%) | 17 (6.8%)  2 (8.0%) | 0.105c  1.000d |
| Pleural space (mm)  early  advanced | 40.5 (36.25-56.5)  34.0 (33.0-34.0) | 35.0 (31.0-44.5)  35.0 (30.0-35.0) | 0.098a  1.000a |
| Collapse rate (%)  early  advanced | 7.0 (4.0-10.0)  6.50 (3.50-9.50) | 7.0 (5.0-10.0)  8.0 (4.0-10.50) | 0.388a  0.674a |
| POD1 effusion (ml)  early  advanced | 250.0 (250.0-262.50)  185.0 (100.0-262.5) | 250.0 (200.0-375.0)  250.0 (200.0-325.0) | 0.289a  0.112a |
| POD2 effusion (ml)  early  advanced | 150.0 (150.0-250.0)  150.0 (50.0-265.0) | 160.0 (100.0-300.0)  150.0 (100.0-250.0) | 0.595a  0.527a |
| POD3 effusion (ml)  early  advanced | 150.0 (150.0-150.0)  150.0 (0.0-300.0) | 150.0 (100.0-200.0)  150.0 (100.0-225.0) | 0.375a  0.541a |
| Chest drainage duration (days)  early  advanced | 3.0 (2.0-4.0)  3.0 (3.0-4.25) | 3.0 (2.0-5.0)  3.0 (2.0-4.0) | 0.078a  0.449a |
| Early complications (pts)  early  advanced | 34 (13.2%)  4 (22.2%) | 45 (17.9%)  7 (28.0%) | 0.139c  0.668c |
| PAL (pts)  early  advanced | 14 (5.4%)  0 (0.0%) | 21 (8.4%)  3 (12.0%) | 0.190c  0.252d |
| Diaphragmatic elevation (yes)  early  advanced | 3 (1.2%)  1 (5.6%) | 4 (1.6%)  0 (0.0%) | 0.721d  0.419d |
| Bronchial angle (°)  early  advanced | 135.0 (121.75-148.25)  141.50 (132.25-148.0) | 125.0 (107.0-146.0)  118.0 (103.0-142.50) | <0.001*a  0.041*a |
| Diaphragmatic paralysis (yes)  early  advanced | 3 (1.2%)  1 (5.6%) | 5 (2.0%)  0 (0.0%) | 0.499d  0.419d |
| Notes: Data are presented as mean (±SD) median (P25–P75) or n (%). *p<0.05. a Mann–Whitney U test. b t-test. c Chi-square test. d Fisher's exact test.  Abbreviations: POD, postoperative day; pts, patients; PAL, prolonged air leak. | | | |
