# Supplementary material for: Impact of Pulmonary Ligament Resection in Upper Lobectomies: A Multicenter Matched Cohort Study
Source: J Clin Med. 2024 Nov 18;13(22):6950. doi: 10.3390/jcm13226950 (PMC11594900; doi:10.3390/jcm13226950)
Supplement: Supplementary file 1 [file jcm-13-06950-s001.zip › Supplementary File.3.docx]

| Supplementary Table S2: Characteristics of Patients of the Shanghai Chest Hospital after PSM | | | |
| --- | --- | --- | --- |
|  | NO LIGAMENT RESECTION (n=130) | LIGAMENT RESECTION (n=0) | p Value |
| Age | 64.0 (56.0-70.0) | - - | - |
| Sex  Male  Female | 71 (54.6%)  59 (45.4%) | - | - |
| Comorbidities (pts) | 64 (49.2%) | - | - |
| Charlson Comorbidity Index | 3.0 (2.0-4.0) | - | - |
| Side  Right  Left | 95 (73.1%)  35 (26.9%) | - | - |
| Surgery Time (minutes) | 112.50 (90.75-121.75) | - | - |
| Estimated blood loss (ml) | 65.0 (50.0-100.0) | - | - |
| Lymph node (number) | 9.0 (6.75-13.0) | - | - |
| Lymph node station (number) | 6.0 (5.0-6.0) | - | - |
| Lymph node station#9 harvested (yes) | 0 (0.0%) | - | - |
| Pleural space (yes) | 21 (16.2%) | - | - |
| Pleural space (mm) | 44.0 (38.5-61.5) | - | - |
| Collapse rate (%) | 6.0 (4.0-11.25) | - | - |
| POD1 effusion (ml) | 25.0 (250.0-250.0) | - | - |
| POD2 effusion (ml) | 150.0 (150.0-150.0) | - | - |
| POD3 effusion (ml) | 150.0 (150.0-150.0) | - | - |
| Chest drainage duration (days) | 3.0 (2.0-4.0) | - | - |
| Discharge with drainage | 0 (0.0%) | - | - |
| Postoperative bronchoscopy abnormalities (yes) | 0 (0.0%) | - | - |
| Bronchial kinking | 0 (0.0%) | - | - |
| LOH (days) | 6.0 (4.0-8.0) | - | - |
| In-hospital mortality | 0 (0.0%) | - | - |
| 30-day mortality | 0 (0.0%) | - | - |
| 90-day mortality | 0 (0.0%) | - | - |
| Early complications (pts) | 13 (10.0%) | - | - |
| PAL (pts) | 2 (1.5%) | - | - |
| Diaphragmatic elevation (yes) | 0 (0.0%) | - | - |
| Clavien-Dindo Classification  Grade 1  Grade 2  Grade 3A  Grade 3B  Grade IVA  Grade IVB  Grade V | 0 (0.0%)  13 (10.0%)  0 (0.0%)  0 (0.0%)  0 (0.0%)  0 (0.0%)  0 (0.0%) | - | - |
| Bronchial angle (°) | 130.0 (115.0-144.0) | - | - |
| Diaphragmatic paralysis (yes) | 0 (0.0%) | - | - |
| Histology  adenocarcinoma  SSC  large cell carcinoma  adenosquamous | 122 (93.8%)  6 (4.6%)  1 (0.8%)  1 (0.8%) | - | - |
| Tumor size (cm) | 2.2 (1.5-2.5) | - | - |
| Tumor status  pT1a  pT1b  pT1c  pT2a  pT2b  pT3  pT4 | 5 (3.8%)  53 (40.8%)  49 (37.7%)  21 (16.2%)  2 (1.5%)  0 (0.0%)  0 (0.0%) | - | - |
| Lymph node status  N0  N1  N2 | 126 (96.9%)  2 (1.5%)  2 (1.5%) | - | - |
| Lymph node station#9 positive | 0 (0.0%) | - | - |
| TNM staging (8th edition)  IA  IB  IIA  IIB  IIIA  IIIB | 106 (81.5%)  18 (13.8%)  2 (1.5%)  2 (1.5%)  2 (1.5%)  0 (0.0%) | - | - |
| 1-year survival | 130 (100%) | - | - |
| Notes: Data are presented as median (P25–P75) or n (%).  Abbreviations: PSM: propensity score matching; BMI, body mass index; FEV1, Forced Expiratory Volume after 1 s; FVC, forced vital capacity, pts, patients, POD, postoperative day; LOH, length of hospital stay, ARDS, acute respiratory distress syndrome; PAL, prolonged air leak; RLN, recurrent laryngeal nerve; ABPP, autologous blood patch pleurodesis; SSC, squamous cell carcinoma. | | | |

| Supplementary Table S3: Characteristics of Patients of the Padua University Hospital after PSM | | | |
| --- | --- | --- | --- |
|  | NO LIGAMENT RESECTION (n=92) | LIGAMENT RESECTION (n=150) | p Value |
| Age | 72.0 (64.0-76.75) | 67.5 (59.0-74.0) | 0.007*a |
| Sex  Male  Female | 52 (56.5%)  40 (43.5%) | 83 (55.3%)  67 (44.7%) | 0.857b |
| Comorbidities (pts) | 80 (87.0%) | 119 (79.3%) | 0.132b |
| Charlson Comorbidity Index | 4.0 (3.0-5.0) | 3.0 (2.0-4.0) | <0.001*a |
| Side  Right  Left | 46 (50.0%)  46 (50.0%) | 88 (58.7%)  62 (41.3%) | 0.188b |
| Surgery Time (minutes) | 125.0 (101.25-143.75) | 139.50 (110.0-160.0) | 0.044*a |
| Estimated blood loss (ml) | 100.0 (100.0-100.0) | 100.0 (100.0-100.0) | 1.000a |
| Lymph node (number) | 8.0 (6.0-10.0) | 7.0 (6.0-9.0) | 0.035*a |
| Lymph node station (number) | 5.0 (4.0-5.0) | 5.0 (4.0-6.0) | 0.991a |
| Lymph node station#9 harvested (yes) | 0 (0.0%) | 73 (48.7%) | <0.001*a |
| Pleural space (yes) | 2 (2.2%) | 3 (2.0%) | 1.000d |
| Pleural space (mm) | 39.5 (37.0-39.5) | 40.0 (35.0-40.0) | 1.000a |
| Collapse rate (%) | 7.0 (5.0-9.0) | 7.0 (5.0-9.0) | 0.874a |
| POD1 effusion (ml) | 250.0 (140.0 (468.75) | 300.0 (200.0-450.0) | 0.142a |
| POD2 effusion (ml) | 250.0 (150.0-380.0) | 250.0 (150.0-352.5) | 0.596a |
| POD3 effusion (ml) | 150.0 (71.25-300.0) | 155.0 (150.0-250.0) | 0.361a |
| Chest drainage duration (days) | 3.0 (2.0-5.0) | 3.0 (2.0-5.0) | 0.518a |
| Discharge with drainage | 0 (0.0%) | 0 (0.0%) | - |
| Postoperative bronchoscopy abnormalities (yes) | 1 (1.1%) | 0 (0.0%) | 0.380d |
| Bronchial kinking | 0 (0.0%) | 0 (0.0%) | - |
| LOH (days) | 5.0 (4.0-7.0) | 5.0 (4.0-6.0) | 0.428a |
| In-hospital mortality | 0 (0.0%) | 0 (0.0%) | - |
| 30-day mortality | 0 (0.0%) | 1 (0.7%) | 1.000d |
| 90-day mortality | 0 (0.0%) | 1 (0.7%) | 1.000d |
| Early complications (pts) | 14 (15.2%) | 22 (14.7%) | 0.907b |
| PAL (pts) | 7 /7.6%) | 14 (9.3%) | 0.644b |
| Diaphragmatic elevation (yes) | 0 (0.0%) | 0 (0.0%) | - |
| Clavien-Dindo Classification  Grade 1  Grade 2  Grade 3A  Grade 3B  Grade IVA  Grade IVB  Grade V | 2 (2.2%)  11 (12.0%)  1 (1.1%)  0 (0.0%)  0 (0.0%)  0 (0.0%)  0 (0.0%) | 2 (1.3%)  17 (11.3%)  2 (1.3%)  1 (0.7%)  0 (0.0%)  0 (0.0%)  0 (0.0%) | 0.924b |
| Bronchial angle (°) | 135.0 (122.75-148.75) | 120.0 (10.6.0-148.0) | 0.004*a |
| Diaphragmatic paralysis (yes) | 0 (0.0%) | 0 (0.0%) | - |
| Histology  adenocarcinoma  SSC  large cell carcinoma  adenosquamous | 64 (69.6%)  21 (22.8%)  7 (7.6%)  0 (0.0%) | 128 (85.3%)  12 (8.0%)  8 (5.3%)  2 (1.3%) | 0.644b |
| Tumor size (cm) | 2.75 (2.0-4.0) | 2.75 (2.0-3.5) | 0.700a |
| Tumor status  pT1a  pT1b  pT1c  pT2a  pT2b  pT3  pT4 | 1 (1.1%)  18 (19.6%)  15 (16.3%)  40 (43.5%)  11 (12.0%)  5 (5.4%)  2 (2.2%) | 3 (2.0%)  56 (37.3%)  27 (18.0%)  37 (24.7%)  12 (8.0%)  13 (8.7%)  2 (1.3%) | 0.024*b |
| Lymph node status  N0  N1  N2 | 80 (87.0%)  7 (7.6%)  5 (5.4%) | 134 (89.3%)  8 (5.3%)  8 (5.3%) | 0.773b |
| Lymph node station#9 positive | 0 (0.0%) | 0 (0.0%) | - |
| TNM staging (8th edition)  IA  IB  IIA  IIB  IIIA  IIIB | 3 (35.9%)  34 (37.0%)  9 (9.8%)  7 (7.6%)  8 (8.7%)  1 (1.1%) | 81 (54.0%)  33 (22.0%)  11 (7.3%)  13 (8.7%)  8 (5.3%)  4 (2.7%) | 0.057b |
| 1-year survival | 92 (100.0%) | 149 (99.3%) | 0.433b |
| Notes: Data are presented as median (P25–P75) or n (%). *p<0.05. a Mann–Whitney U test. b Chi-square test, c t-test, d Fisher's exact test.  Abbreviations: PSM: propensity score matching; BMI, body mass index; FEV1, Forced Expiratory Volume after 1 s; FVC, forced vital capacity, pts, patients, POD, postoperative day; LOH, length of hospital stay, ARDS, acute respiratory distress syndrome; PAL, prolonged air leak; RLN, recurrent laryngeal nerve; ABPP, autologous blood patch pleurodesis; SSC, squamous cell carcinoma. | | | |

| Supplementary Table S4: Characteristics of Patients of the Poznan University of Medical Sciences after PSM | | | |
| --- | --- | --- | --- |
|  | NO LIGAMENT RESECTION (n=0) | LIGAMENT RESECTION (n=49) | p Value |
| Age | - | 67.0 (58.0 (70.5) | - |
| Sex  Male  Female | - | 28 (57.1%)  21 (42.9%) | - |
| Comorbidities (pts) | - | 34 (71.4%) | - |
| Charlson Comorbidity Index | - | 4.0 (4.0 (5.50) | - |
| Side  Right  Left | - | 31 (63.3%)  18 (36.7%) | - |
| Surgery Time (minutes) | - | 110.0 (95.0-135.0) | - |
| Estimated blood loss (ml) | - | 50.0 (50.0-125.0) | - |
| Lymph node (number) | - | 16.0 (11.0-21.5) | - |
| Lymph node station (number) | - | 6.0 (5.0-6.5) | - |
| Lymph node station#9 harvested (yes) | - | 13 (26.5%) | - |
| Pleural space (yes) | - | 4 (8.2%) | - |
| Pleural space (mm) | - | 44.5 (32.75-72.75) | - |
| Collapse rate (%) | - | 8.0 (1.0-13.0) | - |
| POD1 effusion (ml) | - | 250.0 (195.0-250.0) | - |
| POD2 effusion (ml) | - | 150.0 (150.0-180.0) | - |
| POD3 effusion (ml) | - | 150.0 (150.0-150.0) | - |
| Chest drainage duration (days) | - | 2.0 (2.0-4.0) | - |
| Discharge with drainage | - | 0 (0.0%) | - |
| Postoperative bronchoscopy abnormalities (yes) | - | 0 (0.0%) | - |
| Bronchial kinking | - | 0 (0.0%) | - |
| LOH (days) | - | 5.0 (4.0-7.5) | - |
| In-hospital mortality | - | 0 (0.0%) | - |
| 30-day mortality | - | 0 (0.0%) | - |
| 90-day mortality | - | 1 (2.0%) | - |
| Early complications (pts) | - | 11 (22.4%) | - |
| PAL (pts) | - | 5 (10.2%) | - |
| Diaphragmatic elevation (yes) | - | 0 (0.0%) | - |
| Clavien-Dindo Classification  Grade 1  Grade 2  Grade 3A  Grade 3B  Grade IVA  Grade IVB  Grade V | - | 0 (0.0%)  9 (18.4%)  2 (4.1%)  0 (0.0%)  0 (0.0%)  0 (0.0%)  0 (0.0%) | - |
| Bronchial angle (°) | - | 128.0 (111.5-153.5) | - |
| Diaphragmatic paralysis (yes) | - | 0 (0.0%) | - |
| Histology  adenocarcinoma  SSC  large cell carcinoma  adenosquamous | - | 34 (69.4%)  11 (22.4%)  4 (8.2%)  0 (0.0%) | - |
| Tumor size (cm) | - | 3.1 (2.1-3.8) | - |
| Tumor status  pT1a  pT1b  pT1c  pT2a  pT2b  pT3  pT4 | - | 1 (2.0%)  6 (12.2%)  14 (28.6%)  16 (32.7%)  6 (12.2%)  6 (12.2%)  0 (0.0%) | - |
| Lymph node status  N0  N1  N2 | - | 37 (75.5%)  4 (8.2%)  8 (16.3%) | - |
| Lymph node station#9 positive | - | 0 (0.0%) | - |
| TNM staging (8th edition)  IA  IB  IIA  IIB  IIIA  IIIB | - | 17 (34.7%)  11 (22.4%)  4 (8.2%)  9 (18.4%)  7 (14.3%)  1 (2.0%) | - |
| 1-year survival | - | 45 (91.8%) | - |
| Notes: Data are presented as median (P25–P75) or n (%).  Abbreviations: PSM: propensity score matching; BMI, body mass index; FEV1, Forced Expiratory Volume after 1 s; FVC, forced vital capacity, pts, patients, POD, postoperative day; LOH, length of hospital stay, ARDS, acute respiratory distress syndrome; PAL, prolonged air leak; RLN, recurrent laryngeal nerve; ABPP, autologous blood patch pleurodesis; SSC, squamous cell carcinoma. | | | |

| Supplementary Table S5: Characteristics of Patients of the Ospedale Borgo Trento - Verona after PSM | | | |
| --- | --- | --- | --- |
|  | NO LIGAMENT RESECTION (n=54) | LIGAMENT RESECTION (n=54) | p Value |
| Age | 70.0 (63.0-72.25) | 71.0 (64.0-74.0) | 0.489a |
| Sex  Male  Female | 38 (70.4%)  16 (29.6%) | 24 (44.4%)  30 (55.6%) | 0.006*b |
| Comorbidities (pts) | 50 (92.6%) | 28 (51.9%) | <0.001*b |
| Charlson Comorbidity Index | 5.0 (4.0-6.0) | 5.0 (4.0-5.0) | 0.196a |
| Side  Right  Left | 32 (59.3%)  22 (40.7%) | 35 (64.8%)  19 (35.2%) | 0.552b |
| Surgery Time (minutes) | 175.0 (127.50-202.50) | 150.0 (120.0-200.0) | 0.385a |
| Estimated blood loss (ml) | 150.0 (100.0-200.0) | 50.0 (50.0-112.50) | <0.001*a |
| Lymph node (number) | 12.0 (12.0-12.0) | 12.0 (12.0-12.0) | 1.000a |
| Lymph node station (number) | 5.0 (3.0-6.0) | 6.0 (6.0-6.0) | <0.001*a |
| Lymph node station#9 harvested (yes) | 0 (0.0%) | 38 (70.4%) | <0.001*b |
| Pleural space (yes) | 7 (13.0%) | 7 (13.0%) | 1.000b |
| Pleural space (mm) | 33.0 (30.0-35.0) | 30.0 (30.0-35.0) | 0.456a |
| Collapse rate (%) | 6.0 (0.0-10.0) | 6.0 (0.0-10.0) | 0.721a |
| POD1 effusion (ml) | 250.0 (150.0-400.0) | 250.0 (150.0-300.0) | 0.218a |
| POD2 effusion (ml) | 200.0 (100.0-350.0) | 100.0 (100.0-100.0) | <0.001*a |
| POD3 effusion (ml) | 150.0 (50.0-262.50) | 100.0 (50.0-100.0) | 0.003*a |
| Chest drainage duration (days) | 3.0 (3.0-4.0) | 3.0 (3.0-4.0) | 0.935a |
| Discharge with drainage | 0 (0.0%) | 0 (0.0%) | - |
| Postoperative bronchoscopy abnormalities (yes) | 0 (0.0%) | 0 (0.0%) | - |
| Bronchial kinking | 0 (0.0%) | 0 (0.0%) | - |
| LOH (days) | 3.0 (3.0-4.25) | 3.0 (3.0-4.25) | 0.633a |
| In-hospital mortality | 0 (0.0%) | 0 (0.0%) | - |
| 30-day mortality | 0 (0.0%) | 0 (0.0%) | - |
| 90-day mortality | 0 (0.0%) | 0 (0.0%) | - |
| Early complications (pts) | 11 (20.4%) | 11 (20.4%) | 1.000b |
| PAL (pts) | 5 (9.3%) | 3 (5.6%) | 0.462b |
| Diaphragmatic elevation (yes) | 4 (7.4%) | 4 (7.4%) | 1.000b |
| Clavien-Dindo Classification  Grade 1  Grade 2  Grade 3A  Grade 3B  Grade IVA  Grade IVB  Grade V | 4 (7.4%)  6 (11.1%)  0 (0.0%)  1 (1.9%)  0 (0.0%)  0 (0.0%)  0 (0.0%) | 3 (5.6%)  6 (11.1%)  0 (0.0%)  0 (0.0%)  2 (3.7%)  0 (0.0%)  0 (0.0%) | 0.534b |
| Bronchial angle (°) | 148.0 (135.0-150.0) | 123.5 (106.0-134.0) | <0.001*a |
| Diaphragmatic paralysis (yes) | 4 (7.4%) | 5 (9.3%) | 0.728b |
| Histology  adenocarcinoma  SSC  large cell carcinoma  adenosquamous | 34 (63.0%)  11 (20.4%)  0 (0.0%)  9 (16.7%) | 41 (75.9%)  7 (13.0%)  0 (0.0%)  6 (11.1%) | 0.343b |
| Tumor size (cm) | 2.65 (1.7-3.7) | 2.05 (1.1-3.1) | 0.014*a |
| Tumor status  pT1a  pT1b  pT1c  pT2a  pT2b  pT3  pT4 | 7 (13.0%)  17 (31.5%)  14 (25.9%)  6 (11.1%)  1 (1.9%)  9 (16.7%)  0 (0.0%) | 12 (22.2%)  17 (31.5%)  10 (18.5%)  10 (18.5%)  4 (7.4%)  0 (0.0%)  1 (1.9%) | 0.022*b |
| Lymph node status  N0  N1  N2 | 44 (81.5%)  5 (9.3%)  5 (9.3%) | 49 (90.7%)  2 (3.7%)  3 (5.6%) | 0.358b |
| Lymph node station#9 positive | 0 (0.0%) | 2 (3.7%) | 0.153b |
| TNM staging (8th edition)  IA  IB  IIA  IIB  IIIA  IIIB | 37 (68.5%)  0 (0.0%)  0 (0.0%)  10 (18.5%)  7 (13.0%)  0 (0.0%) | 37 (68.5%)  8 (14.8%)  3 (5.6%)  2 (3.7%)  4 (7.4%)  0 (0.0%) | 0.002*b |
| 1-year survival | 52 (96.3%) | 53 (98.1%) | 0.558b |
| Notes: Data are presented as median (P25–P75) or n (%). *p<0.05. a Mann–Whitney U test. b Chi-square test.  Abbreviations: PSM: propensity score matching; BMI, body mass index; FEV1, Forced Expiratory Volume after 1 s; FVC, forced vital capacity, pts, patients, POD, postoperative day; LOH, length of hospital stay, ARDS, acute respiratory distress syndrome; PAL, prolonged air leak; RLN, recurrent laryngeal nerve; ABPP, autologous blood patch pleurodesis; SSC, squamous cell carcinoma. | | | |

| Supplementary Table S6: Characteristics of Patients of the Tor Vergata University Polyclinic after PSM | | | |
| --- | --- | --- | --- |
|  | NO LIGAMENT RESECTION (n=0) | LIGAMENT RESECTION (n=23) | p Value |
| Age | - | 68.0 (55.0-75.0) | - |
| Sex  Male  Female | - | 6 (26.1%)  17 (73.9%) | - |
| Comorbidities (pts) | - | 17 (73.9%) | - |
| Charlson Comorbidity Index | - | 5.0 (3.0-5.0) | - |
| Side  Right  Left | - | 17 (73.9%)  6 (26.1%) | - |
| Surgery Time (minutes) | - | 204.0 (190.0-232.0) | - |
| Estimated blood loss (ml) | - | 50.0 (50.0-50.0) | - |
| Lymph node (number) | - | 8.0 (7.0-11.0) | - |
| Lymph node station (number) | - | 3.0 (2.0-4.0) | - |
| Lymph node station#9 harvested (yes) | - | 3 (13.0%) | - |
| Pleural space (yes) | - | 5 (21.7%) | - |
| Pleural space (mm) | - | 40.0 (33.5-44.5) | - |
| Collapse rate (%) | - | 15.0 (10.0-25.0) | - |
| POD1 effusion (ml) | - | 250.0 (170.0-320.0) | - |
| POD2 effusion (ml) | - | 220.0 (130.0-380.0) | - |
| POD3 effusion (ml) | - | 150.0 (100.0-280.0) | - |
| Chest drainage duration (days) | - | 6.0 (5.0-7.0) | - |
| Discharge with drainage | - | 0 (0.0%) | - |
| Postoperative bronchoscopy abnormalities (yes) | - | 0 (0.0%) | - |
| Bronchial kinking | - | 0 (0.0%) | - |
| LOH (days) | - | 8.0 (7.0-10.0) | - |
| In-hospital mortality | - | 1 (4.3%) | - |
| 30-day mortality | - | 1 (4.3%) | - |
| 90-day mortality | - | 1 (4.3%) | - |
| Early complications (pts) | - | 8 (34.8%) | - |
| PAL (pts) | - | 2 (8.7%) | - |
| Diaphragmatic elevation (yes) | - | 0 (0.0%) | - |
| Clavien-Dindo Classification  Grade 1  Grade 2  Grade 3A  Grade 3B  Grade IVA  Grade IVB  Grade V | - | 1 (4.3%)  4 (17.4%)  1 (4.3%)  1 (4.3%)  1 (4.3%)  0 (0.0%)  1 (4.3%) | - |
| Bronchial angle (°) | - | 130.0 (115.0-148.0) | - |
| Diaphragmatic paralysis (yes) | - | 0 (0.0%) | - |
| Histology  adenocarcinoma  SSC  large cell carcinoma  adenosquamous | - | 19 (82.6%)  4 (17.4%)  0 (0.0%)  0 (0.0%) | - |
| Tumor size (cm) | - | 2.1 (1.4-3.1) | - |
| Tumor status  pT1a  pT1b  pT1c  pT2a  pT2b  pT3  pT4 | - | 0 (0.0%)  11 (47.8%)  4 (17.4%)  6 (26.1%)  1 (4.3%)  1 (4.3%)  0 (0.0%) | - |
| Lymph node status  N0  N1  N2 | - | 20 (87.0%)  2 (8.7%)  1 (4.35) | - |
| Lymph node station#9 positive | - | 0 (0.0%) | - |
| TNM staging (8th edition)  IA  IB  IIA  IIB  IIIA  IIIB | - | 14 (60.9%)  4 (17.4%)  1 (4.3%)  3 (13.0%)  1 (4.3%)  0 (0.0%) | - |
| 1-year survival | - | 23 (100.0%) | - |
| Notes: Data are presented as median (P25–P75) or n (%).  Abbreviations: PSM: propensity score matching; BMI, body mass index; FEV1, Forced Expiratory Volume after 1 s; FVC, forced vital capacity, pts, patients, POD, postoperative day; LOH, length of hospital stay, ARDS, acute respiratory distress syndrome; PAL, prolonged air leak; RLN, recurrent laryngeal nerve; ABPP, autologous blood patch pleurodesis; SSC, squamous cell carcinoma. | | | |
