# Supplementary figures and images for: Impact of Pulmonary Ligament Resection in Upper Lobectomies: A Multicenter Matched Cohort Study
Source: J Clin Med. 2024 Nov 18;13(22):6950. doi: 10.3390/jcm13226950 (PMC11594900; doi:10.3390/jcm13226950)

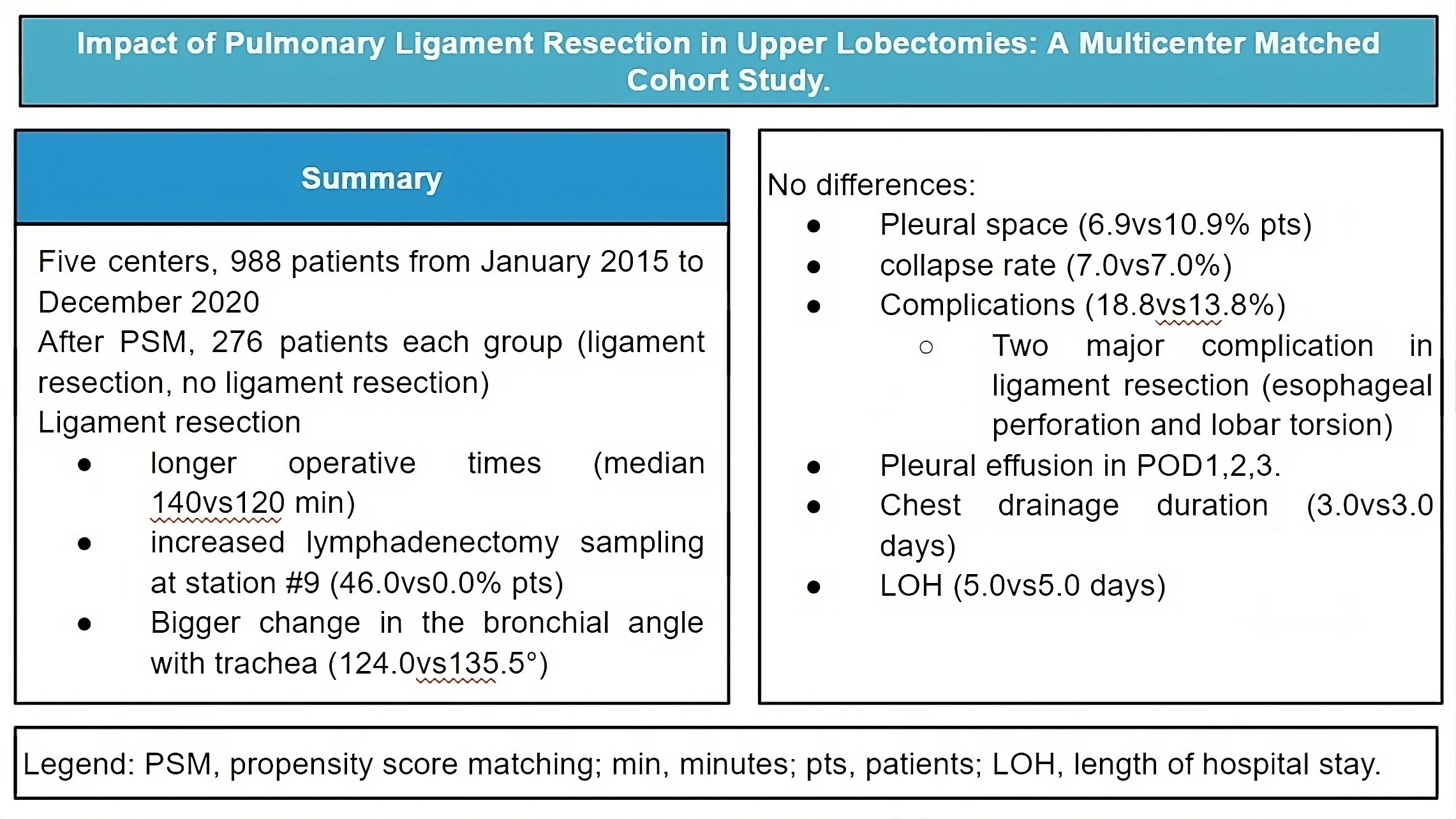

Supplement: Supplementary file 1 [file jcm-13-06950-s001.zip › ligament jcm/graphical-abstract-ligament-jcm.jpg]
