# Supplementary material for: Impact of Pulmonary Ligament Resection in Upper Lobectomies: A Multicenter Matched Cohort Study
Source: J Clin Med. 2024 Nov 18;13(22):6950. doi: 10.3390/jcm13226950 (PMC11594900; doi:10.3390/jcm13226950)
Supplement: Supplementary file 1 [file cover letter ligament.pdf › ligament jcm/cover letter ligament.pdf]

Dear Editor in Chief of the Journal,

I would like to submit a case report entitled “**Impact of Pulmonary Ligament Resection in Upper Lobectomies: A Multicenter Matched Cohort Study.”.**

I confirm that this work is original and has not been published elsewhere, nor is it currently under consideration for publication elsewhere.

In this paper, we compare the outcomes of pulmonary ligament resection vs. non-resection in upper lobectomies for Non-small Cell Lung Cancer.

The authors declare to have no conflict of interest directly or indirectly related to the manuscript contents. The reason for having more than 10 authors in this manuscript is the extensive collaboration involving multiple centers in the study. This collaboration necessitated an intensive and collective effort in collecting, analyzing, and evaluating data and results.

The paper has been prepared to fit as “Article”.

Please address all correspondence concerning this manuscript to me at “campisi.alessio88@gmail.com”.

Thank you for your consideration of this manuscript.

Sincerely, Alessio Campisi
